# Dr. Hunter’s Address on Oral Sepsis

**Published:** 1911-12-15

**Authors:** R. Ottolengui

**Affiliations:** Editor, Items of Interest


### DR. HUNTER'S ADDRESS ON ORAL SEPSIS.

BY R. OTTOLENGUI, EDITOR, ITEMS OF INTEREST.

"Is there any truth in what he said?" or rather in what the *Current Literature* man said that he said, for in the last analysis there is little doubt that our professional brethren have been more angered by what was imputed to Dr. Hunter, than they ever would have been by what he really did say.

The very title of the *Current Literature* article is grossly misleading, and gravely misrepresents Dr. Hunter's position. The original paper is a lengthy disquisition on the subject matter, and discusses the relative fields of general surgery, medical practice and dentistry, though in regard to the last the writer's comments are aimed more particularly at that limited part of dentistry known as "Crown and Bridge-work." Dr. Hunter nowhere denounces either dentistry *per se*, or American dentistry particularly.

After describing the somewhat appalling results which he has noticed as sequellae of ill-constructed and ill-advised bridgework, he mentions sarcastically, "The pride which the dentist responsible for it feels in his high-class' American work.' " The context and the quotation marks clearly indicate that Dr. Hunter is not denouncing American dentistry, but a spurious counterfeit thereof, which is advertised to the confiding public of his country under that name. Later on he does say that, "To gold-cap a healthy or diseased tooth in order to beautify or 'preserve' it is a negation of every one of these truths." Do we deny this? Then he adds that in his observation, in no country is this more common than among Americans, and in America. Is there any libel in that? It is not very long ago that a stage-manager in New York announced that no member of the chorus having conspicuous gold crowns could have place in the front row of his ensembles.

That Dr. Hunter is not "denouncing dentistry," nor the work of "even the best dentists," as has been attributed to him, is abundantly shown in the following quotation:

"The problem is an important one for the dental profession, and its solution an important one in the interests of public health, especially of our school children, 30 to 50 per cent. of whom suffer from dental and oral sepsis, and its tonsillitic, pharyngeal, glandular, and other effects. For while a large body of that profession are engaged in dealing

*successfully* (italics ours) with the difficult problems of dental disease and of oral sepsis, another body is no less steadily engaged in promoting sepsis of the worst character and degree by ignoring the fundamental truths connected with the anatomy, physiology, and pathology of the tissues with which they deal."

No one with any sense of justice can read this and deny that Dr. Hunter has fairly discriminated between the best and the worst practitioners of dentistry. Nor can anyone, in this view of his article, and having recovered from the rancor aroused by the misrepresentations of the sensational writer in *Current Literature*, once again read over, even the passages quoted in that magazine and not admit that Dr. Hunter has not overdrawn his picture; that such instances of dental malpractice are all too common, and therefore Dr. Hunter has done us a kindness rather than an injury in calling attention to it. Even the saffron-hued "review" in *Current Literature* may produce some good, if it but arouse within the bosom of the dear public a wholesome caution in their choice of dental attendants, or a realization of the dangers which may beset them when seated in the chair of the man who advertises that he "inserts teeth without plates."

In any event, as a body of professional men avowedly devoting our lifework to the preservation of the health of our patrons, instead of rushing into print with self-laudatory asseveration, and intemperate invective against Dr. Hunter, would it not be more modest, more dignified, and perhaps more profitable to dispassionately consider the facts in order to determine to what extent this "septic dentistry," as Dr. Hunter terms it, may actually exist?

Dr. Hunter graphically describes the "septic dentistry," which he tells us has been responsible for serious ills in cases which have been observed by him. But elsewhere in his address he charges that the regular medical practitioner is also sadly at fault, or else woefully ignorant in his treatment of patients without due regard to the dangers from sepsis, and especially of oral sepsis. The general surgeon likewise comes in for his share of criticism. Dr. Hunter accords him all credit for his antiseptic precautions in guarding the actual field of his operation. Of the surgeon, he says:

"No precaution is too minute, no duty, however trivial, appears to him as such in the attainment of his great object. He renders these services, he observes these precautions as faithfully and as strictly in the case of the outcast admitted from the poorest slum as he would to the highest personage in the land."

The writer pauses here to ask if this quotation from Dr. Hunter is not an excellent word picture of the truly professional surgeon. Would it not be well to print these words and hang them framed in every dental office as an inspiration to dental surgeons, especially when treating root canals for poor patients?

After this lauding the surgeon for guarding against the introduction of organisms into the body as a result of his operation, he criticizes him for overlooking, or at least shirking responsibility for, organisms that may be already present prior to his operation, especially within the oral cavity. He says:

"And yet unknown to him (the surgeon) and unnoted by him, he may be allowing his patient to retain a degree of sepsis, in the mouth, which would receive his immediate attention were it found anywhere else."

Let us then absolve Dr. Hunter of the charge that he has "denounced American dentistry," and endeavor to measure the extent to which "septic dentistry" may fairly be chargeable at our door.

DOES SEPTIC DENTISTRY EXIST.

That such an investigation cannot but be advantageous is readily shown by a single incident in the history of dentistry in New York City. About ten years ago a prominent physician rather startled the local profession by reading a paper before a body of dentists in which he charged that a seriously large number of persons, within his individual hospital experience, had *died of septic pneumonias*, induced by tooth sockets neglected after extractions. He pointed out that teeth are commonly extracted because of diseased conditions, and that to remove the tooth, and then leave the infected socket to its own resources, is contrary to correct surgical procedure. He made his picture sufficiently convincing to cause a complete revolution in the extracting methods of the dentists of the Metropolis.

Much the same situation confronts us now. Another physician tells of us the evils that he has seen resulting from dental malpractice. What wots it that he may be unfamiliar with the best modern dental methods? The point of importance is that it should ever have been possible for him to have any true foundation whatever for such complaint.

But Dr. Hunter is not the first who has called attention to this condition of affairs. Dentists, even before the surgeons and physicians, made use of the X-rays as an aid to diagnosis, and very quickly we began to "see" things that were as unwelcome as they were unexpected. Long had we proudly believed that we could, and did, fill all root canals to their ends, or at least almost all, almost to their ends. But with the installation of X-ray machines, Morton, Kells, Van Woert, Price, and others began to create a grave doubt on this score.

### ROOT CANAL FILLINGS.

In 1901, on April 1st. (aptly enough), Weston A. Price delivered an illustrated lecture before the Cleveland Dental Society (*Items of Interest*, June, 1901, p. 458-472), demonstrating, "Practical Progress in Dental Skiagraphy." He published 85 radiographs, of which 35 show alveolar abscesses on filled teeth. There are 30 examples of imperfect root fillings which resulted in septic infections; worse yet the majority of these are on single-rooted teeth. Commenting thereon Dr. Price said:

"We will now turn our attention to the dentists' graveyard, root canal fillings, where so many cover up defective, carless work, trusting it will never come to light, and often reminding the patient that when this tooth gives trouble again it will have to be extracted. Humanity should thank God for a new light that will go into these dark places and show up what is often criminally careless or wilfully bad work in filling roots. True, it is often impossible to properly fill roots, but if all were as well filled as possible, those imperfectly filled would be only those with so small a canal or so little of it unfilled that the woes of humanity from this source would be infinitely less than they are."

One would suppose that some improvement in methods would have resulted from such an *expose*, and the more general utilization of radiography, yet in July, 1908, seven years later, Dr. M. L. Rhein delivered a similar illustrated lecture before the New Jersey State Dental Society (*Items of Interest*, November, 1908, p. 833-855). Dr. Rhein published 46 radiographs showing abscesses and other septic sequellae of incorrect root work and improper crown and bridgework. What was of more importance, he described a technique, which, aided by radiography, had enabled him to cure the septic troubles and to correctly treat and fill the roots which previously had been failures in other men's hands. That such roots can be correctly filled was abundantly shown by his radiographs taken after root filling.

In the same issue editorial comment was made upon Dr. Rhein's paper, from which the following quotation may be timely:

"The complete removal of canal contents has been the bugaboo of dentists since dentistry began. Perhaps no single operation has been more written upon, and yet with the hundreds of methods which have been described as adequate, even to-day, if a man dare say that he can reach the apices of even the majority of tooth roots, not a few of his hearers will believe that he is either self-deceived or else a wilful falsifier and a braggart. Yet certainly it is high time, for the honor of American dentistry, that this attitude should pass and that root canal treatment should be as skillfully and as successfully done as are other operations, for there is nothing in dental practice of so great importance. Of what advantage can it be to a patient for a practitioner to insert a perfectly matched porcelain inlay, or a perfectly occluded gold inlay, or a marvelously constructed bridge, if the canals of the supporting teeth have been inefficiently cleansed and filled?

" 'The operation was a success, but the patient died,' is the sneer that is often cast upon the general surgeon. Is it any better to have it said of the dentist: 'His bridge was splendid, but the roots abscessed?' "

On November 9, 1909, Prof. I. N. Broomell delivered an illustrated lecture before the First District Dental Society of New York City, entitled, "The Adventitious Effect of Large Masses of Gold in Contact with Tooth Tissues" (*Dental Cosmos*, April, 1910, p. 389-403.) Dr. Broomell discussed the insertion of large gold fillings near to living pulps, as well as the placing of gold shell crowns without removal of the pulp and he says:

"As a result of the microscopic study of a number of cases, it is proposed to show that almost invariably the pulp tissue, and as well the enamel, dentin, and alveolo-dental membrane, are all more or less affected in various ways by such unnatural surroundings. Among the morbid effects produced are hyperemia of the pulp, dry gangreen of the pulp, calcification of the pulp tissue, sclerosis of the blood vessels of the pulp, atrophy of the pulp, pulp nodules, secondary dentin, expansion of the dentinal tubules, with corresponding bulging of the dentinal fibers, pathologic pigmentation of the enamel and dentin, non-carious destruction of the enamel, as well as destruction by caries and perhaps a molecular change in this tissue. Any one of the foregoing may be the primary affection, after which a train of pathic conditions may ensue."

This paper, by Dr. Broomell, clearly demonstrates by the clinical histories recounted and the beautiful photomicrographs published that serious pathological conditions are to be expected from the all too common practice of "conserving" (sic?) pulps, which should be removed, and which are left in place because the dentist confesses to himself (privately) his inability to aseptically treat and fill the root canals; for which reason he fills, crowns and bridges over doomed or dying pulps, ignorant or else unmindful of just such septic results as are now complained of by Dr. Hunter.

### BRIDGEWORK.

A few months later (*Items of Interest*, October, 1910, p. 754-755) Dr. Herman E. S. Chayes published an article entitled, "Empiricism in Bridgework," in connection with which he published illustrations made from photographs of sixteen pieces of bridgework. Of this class of work he says:

"At best one is brought face to face with results that are unsatisfactory, incomplete, unwholesome and unclean, etc., etc." Of the specimens from which the illustrations are made, he says: "These specimens are not the work of all-around incompetents; authors, clinicians, professors of prosthodontia figure among those at whose doors the existence of these specimens may be laid."

Here, then, we find not a "denunciation of American dentistry by an English physician," but an arraignment of American dental incompetency by four American dentists. A man once asked another, "Why are you so angry because Jones called you a liar?" "That is not what makes me mad," was the reply; "I am mad because he proved it."

The dentists of this country are "mad" because of an article in *Current Literature*, which really misrepresents Dr. Hunter, yet they did not lose their tempers when Drs. Price, Rhein, Broomell and Chayes, not only criticized American dentistry, but proved their contentions with their radiographs, photomicrographs and photographs. The present irritation arises perhaps because of the fact that the latest criticism appeared in the secular press.

It is not the writer's intention to leave the impression that such evils as are here described are representative of typical American dentistry. Quite to the contrary, no one better than he knows that perfectly scientific, thoroughly aseptic, and entirely sanitary dental operations are done daily by thousands of dentists in this country. Yet it is likewise true, and pity 'tis that 'tis true, that thousands of other dentists do exactly that sort of work which Dr. Hunter so aptly terms "septic dentistry."

Perhaps the great underlying reason of this may be found in the fact that neither the surgeon, the physician, the dentist, nor the patients themselves fully realize the dangers that threaten the whole body if the oral cavity be a secret storage place for "oral sepsis." When the people come to understand that their very lives depend upon sound teeth in a clean and healthy mouth, then, and not till then, will they cheerfully pay an adequate fee for competent dental service, and in that day more dentists will practice, because they can then afford to practice thoroughly "aseptic dentistry."—*Items of Interest*, November, 1911.

